# Pre-Operative Digital Templating Aids Restoration of Leg-Length Discrepancy and Femoral Offset in Patients Undergoing Total Hip Arthroplasty for Primary Osteoarthritis

**DOI:** 10.7759/cureus.22766

**Published:** 2022-03-02

**Authors:** Joshua Rui Yen Wong, Marc Gibson, Julian Aquilina, Deovrat Parmar, Padmanabhan Subramanian, Parag Jaiswal

**Affiliations:** 1 Trauma and Orthopaedics, Royal Free Hospital, London, GBR

**Keywords:** osteoarthritis, hip replacement, arthroplasty, femoral offset, leg length inequality

## Abstract

Background

Pre-operative planning and templating is a crucial pre-requisite for total hip arthroplasty (THA). Recently, the use of digital radiography has allowed templating to be digitalised instead of traditional methods involving the use of radiograph transparencies. The primary aim of this study was to compare the accuracy in correction of leg length discrepancy (LLD) and restoring femoral offset in patients undergoing THA for primary osteoarthritis with pre-operative digital templating (PDT) versus conventionalplanning without digital templating.

Methods

This retrospective cohort study compared two groups of patients who underwent THA for primary osteoarthritis. During the period of the year 2020, 56 patients underwent THA with pre-operative digital templating and 50 patients without digital templating. Two independent blinded observers recorded all radiological data.

Results

The digital templated and non-digital templated cohorts were matched for variables including age (mean = 71.8 years vs 70.9 years), pre-operative LLD (-4.9mm vs -5.2mm) and pre-operative offset (41.2mm vs 43.7mm). PDT resulted in correction of LLD to <5mm compared to the contralateral hip in 76.8% of cases, 5-10mm in 21.4% and >10mm in one case (1.8%). The non-digital templated cohort had a LLD of <5mm in 50% of cases, 5-10mm in 28% and >10mm in 22%. Chi-square testing demonstrated these results to be statistically significant (p = 0.002). The mean pre-operative offset in the digital templated group was 40mm and 46mm post-operatively. The non-digital templated cohort had a mean pre-operative offset of 42mm and 36mm post-operatively. Independent t-testing revealed statistical significance of these results (p = 0.05).

Conclusion

PDT leads to an increased likelihood of restoring LLD to <5mm and a significantly increased likelihood of preventing lengthening >10mm. PDT also significantly increases the chance of restoring femoral offset to match the pre-operative native hip. Decreased offset is seen predominantly in the non-digitally templated patients.

## Introduction

Total hip arthroplasty (THA) is the principal surgical treatment for end-stage osteoarthritis of the hip [1]. Other indications include fractures of the femoral neck, femoral head avascular necrosis, hip dysplasia, and inflammatory arthritis [2]. Since the 1960s, THA has been found to be an exceptionally reliable procedure with good clinical outcomes up to 20 years later [3,4]. It is unsurprising that THAs were described as the operation of the century by Learmonth et al. [3].

Post-operative leg length discrepancy (LLD) is a well-recognised complication following THA and is associated with significant patient dissatisfaction. The incidence ranges from 1% to 27% [4]. Patient dissatisfaction with LLD after THA is a common reason for litigation particularly against hip arthroplasty surgeons [5]. Increased limb length is more noticeable to patients than shortening. LLD post-THA varies from 3mm to 70mm [1] with a mean of 3mm to 17mm [6]. Minor LLD (defined as less than 5mm difference) is usually asymptomatic, while most patients with a moderate LLD (defined as 5mm to 10mm difference) have readily manageable symptoms, hence still relatively satisfied. Patients with severe post-operative LLD (defined as more than 10mm difference) may experience significant pain or functional impairment [7].

Another debilitating complication associated with THA, especially in the first few months post-operation, is hip dislocation. As such, an important intra-operative challenge in THA is to correct LLD without compromising hip joint stability [8]. This fine balance is achieved by correcting the hip centre of rotation and femoral offset (the distance from the centre of rotation of the femoral head to a line bisecting the long axis of the femur). Correcting hip centre of rotation and femoral offset is also key for improving abductor muscle function, and consequent gait biomechanics [9].

Pre-operative planning and templating guides intra-operative bone cuts and allows for the appropriate determination of definitive size implants. Surgical planning using pre-operative radiological templates [10] has also been shown to reduce intra-operative peri-prosthetic fracture [11]. Templating was traditionally undertaken using conventional methods involving the use of drawings on radiograph transparencies of magnified implants [12]. Newer methods, involving the use of digital radiography, have been found to be an alternative and are increasingly utilised by surgeons prior to THA [12]. This practice is also supported and recommended by the British Orthopaedic Association and the British Hip Society [13].

Digital radiography uses computer software enabling X-ray magnification to be calculated, which allows templates to be scaled accurately to an appropriate magnification vector. Computer software programs such as TraumaCad provide a reliable method of templating THA, whilst simultaneously yielding a high level of accuracy [12]. The software calculates leg-length radiologically by measuring from the vertex of the lesser trochanter, or tip of the greater trochanter, to either the inferior aspect of the acetabular teardrop, the obturator foramen, or the ischial tuberosity. The required leg-length correction is then calculated and an appropriate femoral neck osteotomy level is established based on the type of prostheses selected [14]. Correction of femoral offset is calculated by measurements obtained from the contralateral hip.

The primary aim of this study is to compare the effect of digital templating with conventional planning (non-digital templating) in the restoration of LLD and femoral offset in patients receiving THA for primary osteoarthritis.

This article was previously presented as a meeting abstract at the 41st SICOT Orthopaedic World Congress, the BHS 2021 Annual Scientific Meeting, and the BOA Virtual Congress 2020 on the 16 September 2021, 10 June 2021 and 25 September 2020, respectively.

## Materials and methods

A total of 106 patients between the ages of 39 and 89 years with symptomatic hip osteoarthritis undergoing THA between January 2019 and January 2020 were selected retrospectively in this study. A cohort of 50 patients underwent conventional planning THA by a combination of eight surgeons, and the other cohort of 56 patients had digital templating performed prior to THA by two surgeons. All operative procedures were carried out by high-volume arthroplasty surgeons experienced in their relevant planning methods. All procedures were performed under spinal or general anaesthesia using either a direct lateral or modified Hardinge approach with the patient in the lateral decubitus position. Study registration and approval was obtained prior to commencement of data collection from the quality governance department of the hospital these procedures were performed.

Inclusion and exclusion criteria

Inclusion criteria consisted of patients undergoing THA for primary osteoarthritis whereas patients who underwent THAs performed for trauma, inflammatory arthritis, and revision arthroplasty were excluded from the study. Patients in both groups (digital templating THA and conventional planning THA) were randomly selected to ensure matching of baseline demographic characteristics including age, gender, pre-operative LLD, and pre-operative femoral offset.

Pre-operative planning and surgical technique

All patients underwent routine pre-operative standing radiographs of the pelvis and proximal femur. In the digital templating group, pre-operative templating was performed by the surgeon on the anteroposterior pelvic radiograph (with a 25.4mm calibration sphere) using the TraumaCad software (Orthocrat Ltd, Petach Tikva, Israel) and an assumed 118% magnification correction as previously established [14]. Templating was used to guide the femoral osteotomy cut, choice of implant size, and implant position. Conventional planning THA was guided by pre-operative traditional radiograph standardised templates. An assessment of appropriate limb length was made intra-operatively via assessing the length of the femur at that level of the knee compared to the contralateral limb. Femoral offset was determined pre-operatively and only modified (increased) if hip stability was a concern. In both treatment groups, intra-operative hip stability was tested clinically through a full range of movement before final selection and implantation of the femoral stem and modular head was made.

Outcomes

**Figure 1:**
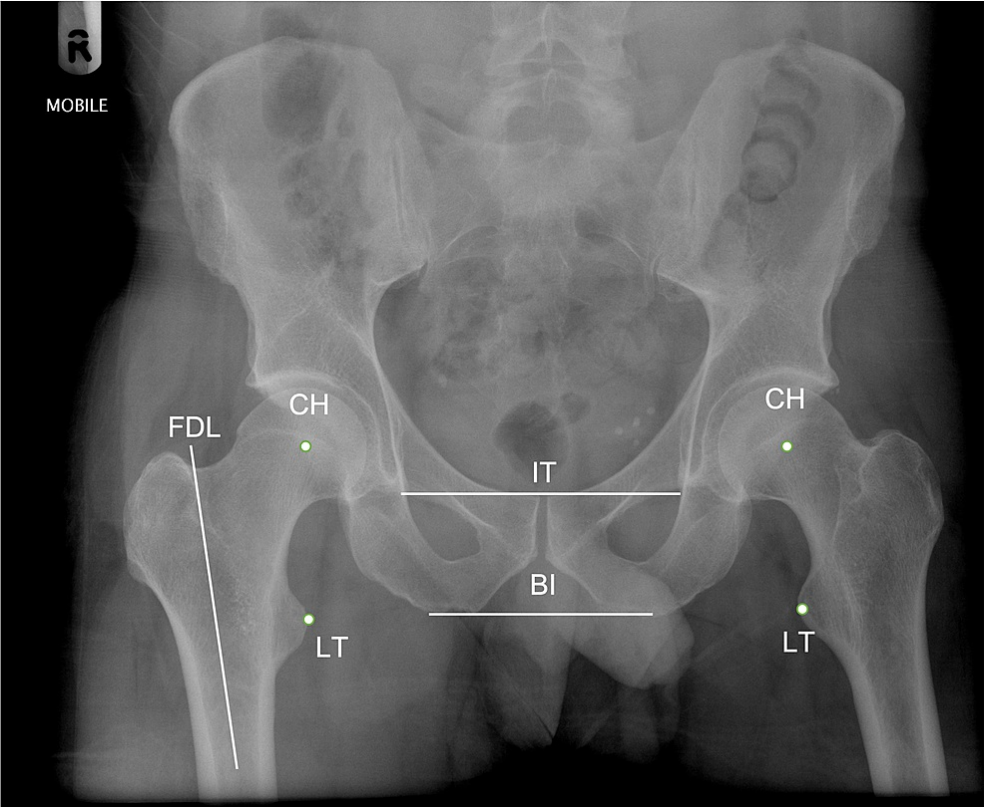
An anteroposterior (AP) radiograph of the pelvis shows the different landmarks marked to measure leg length discrepancy (LLD). CH = center of the femoral head; LT = tip of the lesser trochanter; BI = biischial line; IT = interteardrop line; FDL = Femoral Diaphyseal Line. Original radiograph image courtesy of Dr. Ian Bickle, Radiopaedia.org, rID: 37956

For each patient, post-operative LLD and femoral offset was assessed by two independent reviewers, who were blinded to both the treatment group and each other’s findings. The plain anteroposterior (AP) pelvic radiographs taken three days post-operatively were used to measure the known femoral head size as a reference for correcting magnification. Similarly, LLD was calculated from the plain AP pelvic radiographs. Using the different landmarks shown in Figure 1, LLD was calculated using the vertical distance between the inferior teardrop point and the tip of the lesser trochanter as described by Woolson et al. [15]. Femoral offset was measured horizontally from the centre of the femoral head to the femoral diaphyseal line.

## Results

Patient cohort comparisons

**Figure 2:**
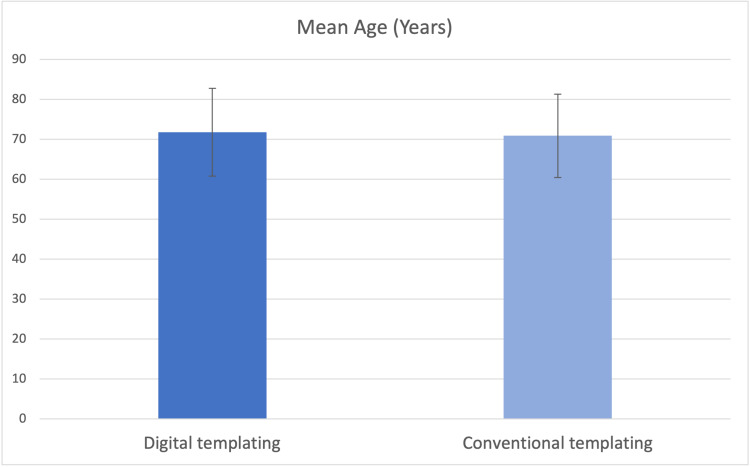
Mean age with SD (arrow bars) in both cohorts.

Three pre-operative variables (age, LLD, and femoral offset) were used to compare both patient groups prior to intervention in order to reduce treatment bias. The digital templated and conventional planning cohorts were shown to be similarly matched with regards to these variables. As seen in Figure 2, the mean age in the digital templated cohort was 71.8 years (standard deviation (SD) of 11 years) while the mean age in the conventional planning cohort was 70.9 years (SD 10.4).

**Figure 3:**
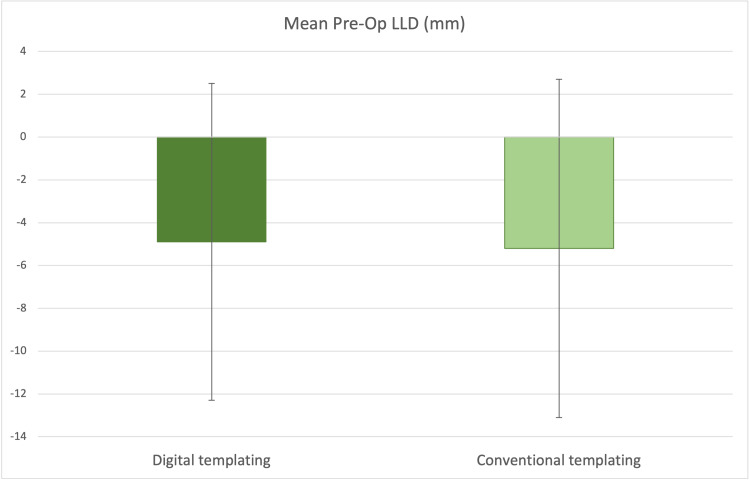
Mean pre-operative leg length discrepancy (LLD) with SD (arrow bars) in both cohorts.

In the digital templated cohort, the mean pre-operative LLD was -4.9mm (SD 7.4mm) when compared to the contralateral limb. In comparison, the conventional planning cohort had a mean pre-operative LLD of -5.2mm (SD 7.9mm) as shown in Figure 3.

**Figure 4:**
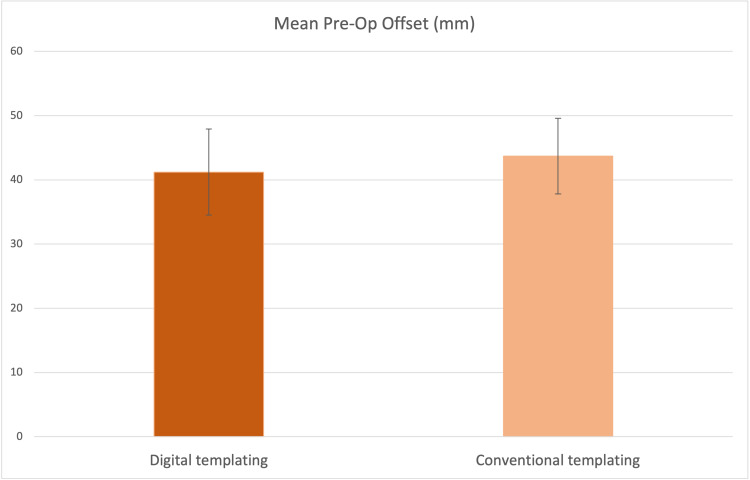
Mean pre-operative femoral offset with SD (arrow bars) in both cohorts.

Pre-operative femoral offset was also similar between both groups (Figure 4).

The digitally templated group had a mean pre-operative offset of 41.2mm (SD 6.7mm). The conventional planned group had a mean pre-operative offset of 43.7mm (SD 5.9mm). Varying modes of THAs were performed with hybrids being the preferred choice as illustrated in Table 1.

**Table 1:** Type of total hip arthroplasty (THA) prosthesis in the two patient groups

	Digitally templated	Conventional planning
Uncemented	9 (16%)	16 (32%)
Hybrid	32 (57%)	22 (44%)
Cemented	15 (27%)	12 (24%)
Total	56	50

Leg-length discrepancy

**Figure 5:**
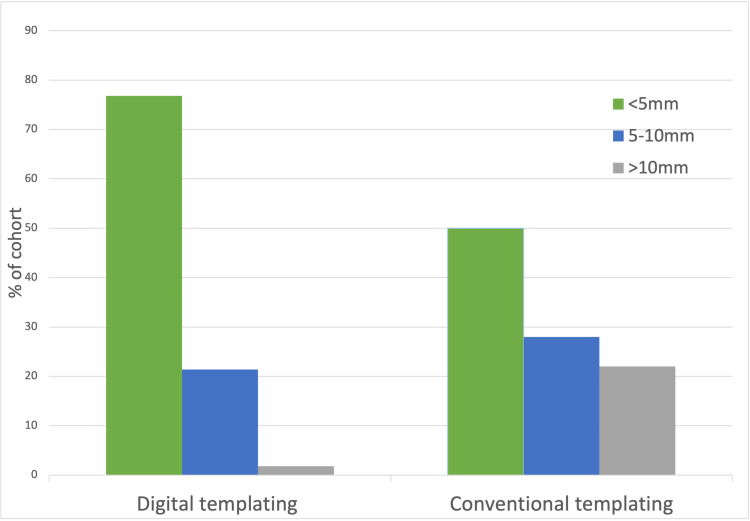
Leg length discrepancy (LLD) post-total hip arthroplasty (THA) in both groups when compared to contralateral limb.

In the cohort of patients where pre-operative digital templating was performed, a correction of LLD to less than 5mm difference from the contralateral limb was achieved in 76.8% of cases (Figure 5). Correction to 5-10mm was achieved in 21.4% of cases, and a LLD of greater than 10mm was observed in 1.8% of cases (one patient). In the cohort where digital templating was not performed, correction of LLD to less than 5mm of the contralateral limb was achieved in 50% of cases, correction to 5-10mm achieved in 28% of cases, and a LLD greater than 10mm observed in 22% of cases (11 patients). Chi-square testing was performed on these measurements demonstrating these results to be statistically significant with a p-value of 0.002.

Femoral offset

**Figure 6:**
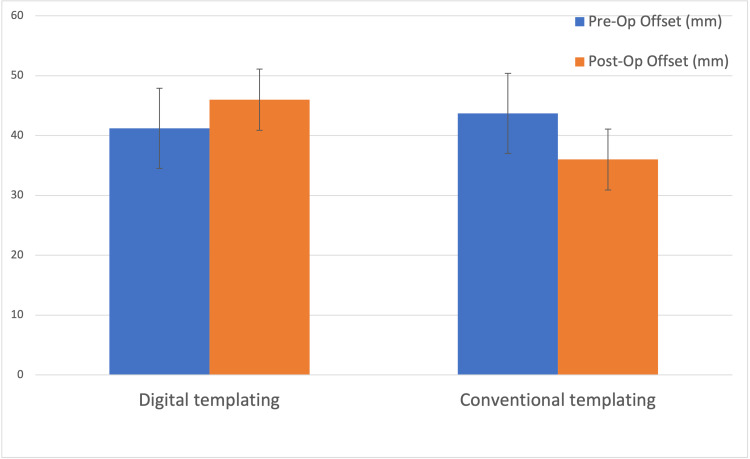
Femoral offset post-total hip arthroplasty (THA) when compared to pre-operative hip.

In the digital templated cohort, mean pre-operative offset was 40mm (SD 4.6mm), with post-operative offset increasing to 46mm (SD 5.1mm). In the conventional planning cohort, mean pre-operative offset was 42mm (SD 8.2mm), with mean post-operative offset reduced to 36mm (SD 6.2mm) as seen in Figure 6. Independent t-testing of these measurements demonstrates statistical significance at a p-value of 0.05.

## Discussion

The two main findings from this study are that pre-operative digital templating leads to improved radiological restoration of LLD and femoral offset in patients undergoing THA. Restoration of LLD is an important objective of THA as it can greatly affect the functional outcome and post-operative pain in the contralateral hip and lower back. Individuals also report negative stigmata from the cosmetic effects of a LLD discrepancy gait. Although absolute correction of leg-length may not be an achievable goal, there is a proposed limit between acceptable and unacceptable levels of LLD. Multiple studies in the literature have reported that up to 10mm of LLD is well tolerated by most patients [16,17]. Marked post-operative LLD may result in significant disability due to pain or functional impairment, occasionally warranting revision surgery [7]. In our study, the digital templated cohort had a higher likelihood of achieving LLD to less than 5mm (77% vs 50%), and a greatly reduced likelihood of significant LLD of more than 10mm (1.8% vs 22%) when compared to the conventional planning THA cohort. These findings are consistent with other studies reporting reliability and a high level of accuracy of digital templating at predicting the required femoral and acetabular implant size, the appropriate bone cuts to be performed, therefore correcting pre-operative LLD [12]. Conversely, there are also histological studies which document superior accuracy using conventional planning methods compared to digital templating in THA [18,19]. Specifically, Iorio et al. reported reduced accuracy of the estimation of the acetabular cup and femoral stem size with digital templating [19]. While González Della Valle et al. found similar accuracy in terms of the femoral stem size, and the precision for predicting acetabular cup size was superior using conventional planning techniques compared to digital templating [18]. Nevertheless, both these studies have reported that digital templating is not only appropriate to determine the sizes of the implants but also acceptably safe in guiding optimal implant positioning [18,19]. In general, digital templating software has improved considerably over the years, and since the time of these abovementioned historical studies [18,19].

The second outcome investigated was femoral offset. Our study results illustrate a statistically significant increase in femoral offset following THA in patients who underwent digital templating pre-operatively compared to conventional planning. Biomechanically, increasing femoral offset is beneficial due to creation of a greater moment arm [20,21], resulting in a reduced abductor muscle requirement and strain, therefore, reducing abductor failure and a subsequent Trendelenburg gait. Notably, other studies have also shown greater abductor strength and a reduced risk of Trendelenburg positive patients with increasing femoral offset [22,23]. Clinical improvements were also observed in another study which demonstrated that female patients with an increased offset femoral stem had a significantly better clinical outcome (Oxford Hip Score at 5 years). Multiple other studies have further investigated the relationship of increased femoral offset with hip function post-operatively, including improved range of movement [22,24] and polyethylene wear [25]. Most recently, Hu et al. quantified the effect of femoral offset difference post-THA [26]. They reported that an appropriate increase of 2-3mm in femoral offset could improve the recovery of hip abduction and external rotation function [26]. Consequently, an optimal femoral offset calculated pre-operatively via digital templating, rather than blindly increasing this distance, could reduce muscle imbalance, gait instability, and THA dislocations [26].

Study limitations

The findings of this retrospective study must be considered in light of some limitations. In terms of study design, our results and conclusions are somewhat limited due to a small sample of 106 patients. Furthermore, the surgeons who performed digital templating only performed THAs on their group of patients, therefore raising the possibility that the abilities of different surgeons between the two cohorts may have had an effect on the outcome measures. In terms of study outcomes, the clinical relevance of LLD or femoral offset changes have not been analysed by measuring validated hip functional outcome scores such as the Oxford or Harris hip scores. Although patient-reported outcomes were not the primary objective of our study, they are a necessary aspect of treatment evaluation especially when comparing different treatments on disability [27].

Another limitation of this study is that LLD measurement is complex and not simply radiographic [28]. Not only does LLD have two differing definitions (true LLD and functional LLD), there are also multiple different radiological and clinical methods to measure LLD. In this study, LLD was measured using radiography and whilst this is the current gold standard, these measurements do not account for the influence of dynamic lower limb malalignment on limb length [29]. Measurement of LLD may be affected by the sagittal and coronal balance of the spine, pelvic tilt, soft tissue contractures, knee and ankle deformities. It should also be considered that templating software such as TraumaCad calculates radiological leg-length by identifying key structures such as the lesser trochanter. These measurements will inevitably be affected by the quality of radiographs, specifically, if rotation of the film is present. Additionally, an external or internal rotated femur will similarly affect the projection of the lesser trochanter on a 2-dimensional film. Essentially, LLD measurement is a complex phenomenon with multiple factors that should be taken into consideration. Nevertheless, we believe the primary aim of the paper has been fulfilled and the utility and effectiveness of digital templating in restoring leg length and femoral offset is clearly evident.

## Conclusions

Given the accessibility and increasing use of digital templating globally, it is important to compare its accuracy with conventional templating in correcting LLD and restoring femoral offset in patients undergoing THA. The findings from this retrospective cohort study suggest that pre-operative digital templating leads to a superior outcome post-THA in terms of restoring LLD and femoral offset. Digital templating also resulted in an increase in femoral offset, however further studies are required to supplement the limited literature concluding the clinical benefits of increased femoral offset post-THA.
